# IL-1 does not reverse the anti-proliferative effect of aspirin in breast cancer cells

**DOI:** 10.1186/1477-7800-3-24

**Published:** 2006-09-04

**Authors:** Paula Sali, Andrew Paul Jewell

**Affiliations:** 1 School of Life Science, Kingston University, Surrey KT1 2EE, UK; 2 Faculty of Health and Social Care Sciences, Kingston University and St George's University of London, Surrey KT1 2EE, UK

## Abstract

**Objectives:**

Interleukin- 1 (IL-1) is a multifunctional proinflammatory cytokine. There have been studies suggesting a role in affecting growth and invasiveness of malignant breast cells by either blocking or stimulating growth of cultured MCF-7 breast cancer cells. This effect may be mediated by induction of COX-2. Aspirin is an inhibitor of COX-2 and has been implicated, with other non-steroidal anti-inflammatory drugs (NSAIDS) in prevention and treatment of breast cancer. In this study the in vitro effects of IL-1 and aspirin on growth of MCF-7 human breast cancer cells was examined.

**Methods:**

MCF-7 cells were treated with various concentrations of IL-1 and aspirin alone and in combination. Cell growth was assessed by cell number measurement.

**Results:**

Aspirin significantly decreased growth rate in a dose-dependant manner, alone and as a combined treatment with IL-1 with a maximum reduction in growth rate at 300 mg/ml (P < 0.05). Treatment with IL-1 alone showed no significant effect on growth rate of MCF-7 cells (P > 0.05).

**Conclusion:**

This study confirms that aspirin suppresses the proliferation rate of MCF-7 cells both as a single agent and in combination with IL-1. It also suggests that IL-1 alone does not stimulate or inhibit growth of MCF-7 cells.

## Background

Interleukin-1 (IL-1) is a pro-inflammatory cytokine affecting many cell types [1,2]. IL-1 is also present in high levels at tumor sites where it has been shown to have effects on the growth and invasiveness of malignant cells by either inducing growth factors that promote carcinogenesis and tumor metastasis [3], or by decreasing tumor growth by direct cytotoxic action [4]. It is thought that expression of high levels of IL-1 cause broad inflammation which damages tissue and therefore tumor invasiveness is induced [1,3]. In addition, IL-1 induce the expression of pro-inflammatory enzymes mainly Cyclooxygenase 2 (COX-2), inducible nitric oxide (iNOS), as well as other cytokines and chemokines. There is also evidence that IL-1 is secreted by various human tumor cells and in most cases increases invasive patterns in the tumor cells; antitumor effects were rarely observed [3]. 90% of invasive breast cancers showed IL-1 expression but to a lesser extent in benign tumors. Furthermore high levels of IL-1 were associated with other parameters of aggressive tumors such as estrogen receptor negativity and p53 gene mutations [3].

Cyclooxygenase (COX) is an enzyme that catalyses the conversion of arachidonic acid to important biological mediators known as prostanoids (prostaglandins, thromboxanes and prostacyclin) [5,6]. COX-2 is an inducible form which is not detected in most healthy tissues but can be induced in response to a variety of stimuli such as proinflammatory cytokines (TNF, IL-1), hormones, bacterial polysaccharides and growth factors [5,7,8]. Studies over recent years have suggested an important role of COX-2 in tumor formation and progression. Various human malignancies including breast cancer have shown increased expression of COX-2. In one study, intermediate or high levels of COX-2 were detected in 43% of Human Breast cancer carcinomas and in 62.5% of Ductal Carcinoma *in Situ*, and also COX-2 expression was found in MCF-7 breast cancer cell line induced by *HER2 *and no COX-2 proteins in MCF-7 cells alone [9]. Davies *et al *also detected COX-2 protein expression in 63 of 89 breast tumors using immunohistochemical analysis [7]. Costa and colleagues revealed that COX-2 was expressed in 17.4% of breast carcinomas studied, analyzed by both immunohistochemistry and western blotting and that COX-2 was not detected in normal breast tissue. The study also showed that COX-2 expression was associated with angiogenesis, lymph node metastasis and apoptosis in human breast cancer [5].

NSAIDs are widely used in treating symptoms such as pain and fever associated with inflammation. Epidemiological studies report that these drugs particularly aspirin may be associated with reduced risk of cancer development. Some studies have focused on reduced incidence of colorectal cancer with NSAIDs use [10], while others have related to breast cancer. One large prospective cohort study by Johnson *et al *reported a trend of decreasing risk of incident breast cancer among postmenopausal women with frequent use of aspirin but not with other NSAIDs [11]. Another case-control study suggested a 40% reduction in breast cancer incidence in women who reported daily intake of aspirin and other NSAIDs for at least ten years [12]. However some studies found no difference in aspirin intake among women who developed breast cancer and those who did not [13]. The main target of NSAIDs is the COX enzyme and acts by efficiently blocking its activity and thus inhibiting COX2. This therefore inhibits production of several prostaglandins which when elevated may promote carcinogenesis, through increased cell proliferation, immune suppression, tumour promotion and facilitate metastasis [11]. Inhibitory effects by certain COX-2 inhibitors on growth in cancer cell lines have been shown in some studies. DFU, [5,5-dimethyl-3-(3-fluorophenyl)-4-(4-methylsulphonyl)phenyl-2(5*H*)-furanone], a selective COX-2 inhibitor was found to significantly reduce tumour growth in mice injected with MCF-7 cells and also inhibited growth when MCF-7 cells were cultured for a long period at high drug concentrations [6]. A study by Goel *et al *found that aspirin inhibited growth in three colon cancer cell lines [14]. Aspirin and its parental compound acetylsalicylic acid causes irreversible inactivation of COX enzyme acting on both COX-1 and -2. In addition to its anti-inflammatory action, aspirin has been known to have anti-proliferative effects as well as anti-apoptotic effects on cancer. This study, therefore aimed to investigate the interactions between the pro-inflammatory cytokine IL-1 and anti-inflammatory aspirin on MCF-7 breast cancer cells.

## Methods

MCF-7 cells were cultured in DMEM with 10% foetal calf serum. Cells were trypsinized, and 2.0 × 10^5 ^cells/ml plated per well in multi well plates. After incubation for twenty four hours, the cells in ten wells from each plate were treated in duplicate with different concentrations of IL-1 or aspirin alone and in combination. Viable cells were counted at 48, 72, 120 and 168 hours and results recorded. Results were analyzed by one-way analysis of variance test (ANOVA).

## Results

### Aspirin inhibited growth of human breast cancer cell line MCF-7

**Figure 1:**
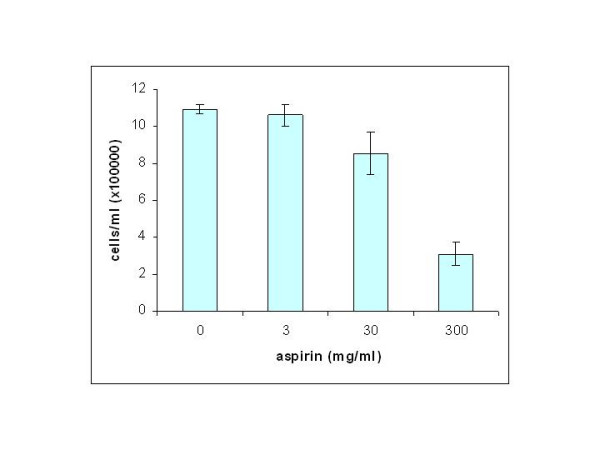
Dose- dependant effects of aspirin on the growth rate of human breast cancer cell line MCF-7. Mean ± SD, n = 2.

Aspirin treatment induced a significant reduction in the cell proliferation rate of the breast cancer cell line as indicated by the viable cell count for each concentration after 120 hours of incubation (Figure 1). At 300 mg/ml the growth inhibitory effect of aspirin was most prominent causing a 70% reduction in cell number compared with cells incubated with medium alone (P < 0.05). A significant reduction of about 20% in proliferation rate was also seen at a concentration as low as 30 mg/ml (P < 0.05). The data establish that aspirin alone can inhibit the growth or proliferation rate of MCF-7 breast cancer cell line.

### No effect of IL-1 on growth of MCF-7 cell line

**Figure 2:**
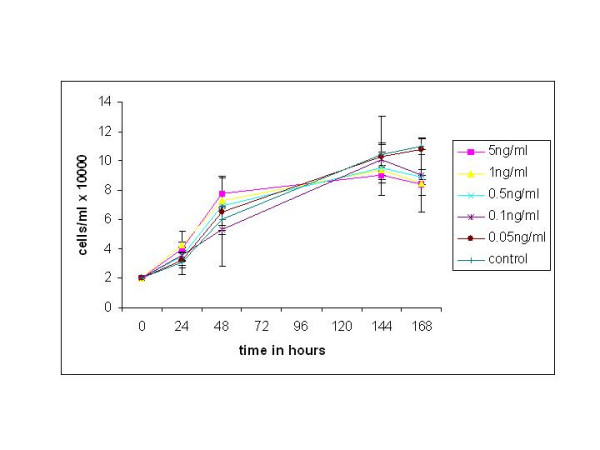
Growth curve for MCF-7 breast cancer cell line exposed to different concentrations of the cytokine IL-1 and incubated for up to 168 hours. Cell counts were performed at 24 h, 48 h, 144 h and 168 h. Mean ± SD, n = 2.

MCF-7 cells were grown in increasing concentrations of IL-1, and cell growth determined by counting viable cells at 24, 48, 72, and 120 hours. No significant decrease in cell growth was observed with concentrations of 5.0-0.1 ng/ml IL-1 compared to the control group after 68 hours of incubation (Figure 2). Therefore this evidence suggests that IL-1 has no significant effect on the growth rate of MCF-7 breast cancer cells alone.

### No effect of IL-1 on aspirin-mediated inhibition of proliferation of MCF-7 cells

**Figure 3:**
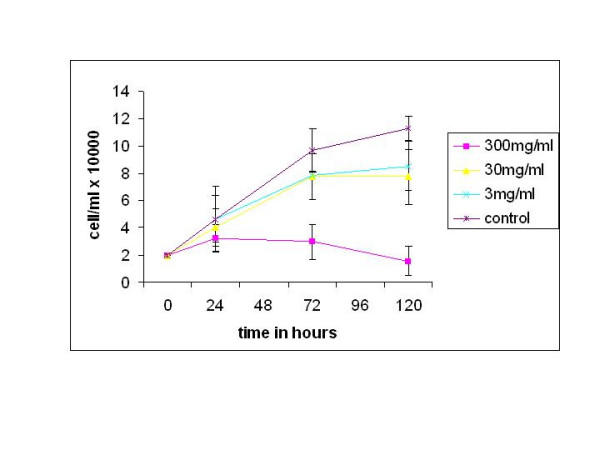
Dose and time- dependant effects of aspirin on the growth of MCF-7 Breast cancer cell line after treatment with IL-1. Cells were treated with 5 ng/ml IL-1, exposed to various concentrations of aspirin and incubated for up to 120 hours. Mean ± SD, n = 2.

MCF-7 breast cancer cells were exposed to 5 ng/ml IL-1 followed by three concentrations of aspirin, incubated and viable cells counted for a period of 72 hours. At 300 mg/ml aspirin, a significant reduction in proliferation rate of about 70% was observed (p < 0.05) compared to the control group, and was also significantly different from the other treatment concentrations after 72 hours (Figure 3). Maximal effect of aspirin inhibition was observed after 120 hours at a dose of 300 mg/ml (p < 0.05). Significant inhibition effects were also seen at 3 mg/ml and 30 mg/ml after 120 hours (p < 0.05).

These findings suggest that aspirin is capable of inhibiting growth rate of MCF-7 cells even when combined with IL-1.

## Discussion

In this study, the effects of the pro-inflammatory cytokine IL-1 and aspirin, a COX-2 inhibitor, on the growth of MCF-7 breast carcinoma cell line *in vitro *were examined. No effect of IL-1 alone on cell proliferation was seen. This reflects the results of previous studies where, IL-1 alone did not induce a significant effect, but an effect was seen only when the cancer cells were cultured with growth factors before treatment with IL-1. The growth of MCF-7 breast cancer cells is controlled by a number of factors such as estrogen and growth factors [15]. Two previous studies showed that IL-1 inhibited growth effects of MCF-7 cells that were stimulated by growth factors, insulin- like growth factor-I (IGF-I) and insulin by targeting and inhibiting the insulin growth factor receptors [4,15]. Both studies also showed that IL-1 alone did not directly induce cell death or impair cell growth. Aspirin and other related NSAIDs have been shown to have chemopreventive activity on cancer [11] as well as inhibiting growth and/or formation of tumors [6] through targeting action of COX-2, an enzyme responsible for production of prostaglandins. COX-2 has been implicated in the development and progression of several tumors. The known mechanisms of the activity of aspirin and other COX-2 inhibitors involve decrease in tumor cell activity and/or proliferation through apoptosis and decreased production of prostaglandins. Prostaglandins such as PGE2 are known to suppress the immune system through down- regulation of lymphokines, proliferation of T and B cells, as well as cytotoxic activity of natural killer cells [16]. In the present study, aspirin showed a markedly response by directly inhibiting growth of the MCF-7 cells, with a maximum effect at a dose concentration of 300 mg/ml. It has been known that COX-2 enzyme is present in many inflamed and neoplastic tissues and that it can be induced in response to variety of stimuli such as growth factors (Vascular Endothelial Growth Factor VEGF, Epidermal Growth Factor EGF, Fibroblast Growth Factor FGF) and pro-inflammatory cytokines (IL-1, TNF) [16]. However, in the present study, no effect of IL-1 on the growth-inhibitory properties of aspirin were seen, and suggests that aspirin mediates the effect directly on breast cancer cells and not through anti-inflammatory pathways.

## References

[B1] Saijo Y, Tanaka M, Miki M, Usui K, Suzuki T, Maemondo M, Hong X, Tazawa R, Kikuchi T, Matsushima K, Nukiwa T (2002). Proinflammatory Cytokine IL-1β Promotes Tumor Growth of Lewis Lung Carcinoma by Induction of Angiogenic Factors: In Vivo Analysis of Tumor-Stromal Interaction. J Immunol.

[B2] Dinarello C (1997). Interleukin-1. Cytokine and Growth Factor Reviews.

[B3] Apte RN, Voronov E (2002). Interleukin-1- a major pleitropic cytokine in tumor- host interactions. Cancer biology.

[B4] Costantino A, Vinci C, Mineo R, Frasca F, Pandini G, Milazzo G, Vigneri R, Belfiore A (1996). Interleukin-1 Blocks Insulin and Insulin Growth Facto-Stimulated Growth In MCF-7 Human Breast Cancer Cells by Inhibiting Receptor Tyrosine Kinase Activity. Endocrinology.

[B5] Costa C, Soares R, Reis-Filho SJ, Leitao D, Amendoeira I, Schmitt FC (2002). Cyclo-oxygenase-2 Expression is associated with angiogenesis and lymph node metastasis in human breast cancer. J Clin Pathol.

[B6] Matsumoto G, Rahman MA, Muta M, Nakamura T, Bando H, Saji S, Tsuruta K, Okamoto A, Toi M (2002). DFU, a selective COX-2 inhibitor, suppresses MCF-7 xenograft tumor growth in mice. Oncology Reports.

[B7] Davies G, Martin LA, Sacks N, Dowsett M (2002). Cyclo-oxygenase-2 (COX-2), aromatase and breast cancer: a possible role of COX-2 inhibitors in breast cancer chemoprevention. Annals of Oncology.

[B8] Ristimaki A, Simula A, Lundin J, Lundin M, Salminen T, Haglund C, Joensuu H, Isola J (2002). Prognostic Significance of Elevated Cyclooxygenase-2 Expression in Breast Cancer. Cancer Research.

[B9] Half E, Tang XM, Gwyn K, Sahin A, Wathen K, Sinicrope FA (2002). Cyclooxygenase-2 Expression in Human Breast Cancers and Adjacent Ductal Carcinoma *in Situ*. Cancer Research.

[B10] Thun MJ, Henley SJ, Patrono C (2002). Nonsteroidal Anti-inflammatory Drugs as Anticancer Agents: Mechanistic, Pharmacology, and Clinical Issues. Journal of the National Cancer Institute.

[B11] Johnson TW, Anderson KE, Lazovich DL, Folsom AR (2002). Association of Aspirin and Nonsteroidal Anti-inflammatory Drug Use with Breast Cancer. Cancer Epidemiology, Biomarkers and Prevention.

[B12] Harris ER, Namboodiri KK, Farrar WB (1996). Nonsteroidal anti-inflammatory drugs and breast cancer. Epidemiology.

[B13] Egan MK, Stampfer MJ, Giovannucci, Rosner BA, Colditz GA (1996). Prospective study of regular aspirin use and the risk of breast cancer. Journal of National Cancer Institute.

[B14] Goel A, Chang DK, Ricciadielllo L, Gashe C, Boland RC (2003). A Novel Mechanism for Aspirin-mediated Growth Inhibition of Human Colon Cancer Cells. Clinical Cancer Research.

[B15] Shen W-H, Zhou J-H, Broussard SR, Freund GG, Dantzer R, Kelly KW (2002). Proinflammatory Cytokines Block Growth of Breast Cancer Cells by Impairing Signals from a Growth Factor Receptor. Cancer Research.

[B16] Gasparini G, Longo R, Sarmiento R, Morabito A (2003). Inhibitors of Cyclo-oxygenase 2: a new class of anticancer agents?. Lancet Oncology.

